# Notification

**DOI:** 10.1111/jch.14712

**Published:** 2023-08-18

Ortiz, M.S. (2022), Inconsistencies in the sensitivity and specificity values in a review paper published in *Journal of Clinical Hypertension* 24: 1390−1391. https://doi.org/10.1111/jch.14353.

This note is for the above Letter to the Editor, published online on August 21, 2021 on Wiley Online Library (wileyonlinelibrary.com), and has been published by agreement between the Journal's Editor‐in‐Chief, Dr Ji‐Guang Wang, and Wiley Periodicals, LLC. In the above Letter to the Editor, concerns were raised regarding inconsistencies in the sensitivity and specificity values in an article by Morisky et al. published in the *Journal of Clinical Hypertension*.^1^ Following an investigation by the Journal, which included an independent statistical review, it was concluded that the results of the article^1^ were misleading due to issues regarding the sensitivity and specificity of the medical adherence scale used. As a result, the Journal no longer has confidence in the reported conclusions and has retracted the article. The Journal is issuing this note to alert readers that the article to which this Letter to the Editor refers has now been retracted.
